# Preparation and Application of Cellulose-Based Thermosensitive Polymer in Water-Based Drilling Fluid

**DOI:** 10.3390/polym18101187

**Published:** 2026-05-12

**Authors:** Xiaodong Bai, Zeyu Xue, Moubo Wang, Molin Song, Mengqian Yang, Jianpeng You, Yumei Luo

**Affiliations:** 1 School of New Energy and Materials, Southwest Petroleum University, Chengdu 610500, China; 2 Sichuan Sizhong Basalt Fiber Technology R&d Co., Ltd., Dazhou 635000, China

**Keywords:** temperature responsive polymer, cellulose, wellbore stability, drilling fluid, plugging agent

## Abstract

The temperature-sensitive plugging agent (HAAN) was synthesized via free-radical graft polymerization using hydroxypropyl methyl cellulose (HPMC), acrylamide (AM), 4-acryloylmorpholine (ACMO) and N-vinyl-2-pyrrolidone (NVP) as the main monomers. HAAN demonstrates potential for addressing the frequent lost circulation problems encountered during the drilling of complex formations. The target product was characterized by FTIR and ^1^H NMR. Its phase transition behavior was verified via temperature-dependent UV-visible transmittance measurements and high-temperature rheological tests. The experimental results show that the plugging agent possesses good temperature and salt tolerance, and its rheological properties can be enhanced by incorporation into drilling fluid. It demonstrates effective plugging performance both at room temperature and under high-temperature conditions, thereby contributing to improved wellbore stability. It provides a new idea for green multifunctional application of cellulose in water-based drilling fluid.

## 1. Introduction

Drilling engineering serves as the primary method for underground resource exploration and extraction, forming a critical foundation for the development of oil and gas resources. Globally, annual upstream investment in oil and gas totals hundreds of billions of US dollars, with drilling and completion costs accounting for 30% to 50% of the total oilfield development costs [1]. However, wellbore instability remains a persistent technical challenge that plagues oil and gas drilling operations worldwide. In recent years, as exploration and development activities intensify, the focus has gradually shifted from conventional to unconventional oil and gas resources. Consequently, advancing technology for deep resource development has become a priority for the industry [2]. This necessitates technological innovation and advancement across exploration and production operations, particularly to address the challenges inherent in developing complex reservoirs [3]. Unconventional gases, particularly shale gas, are playing an increasingly prominent role in China’s natural gas development and production [4,5]. The increasing number of deep and ultra-deep wells in complex geological formations is expanding the scale of shale gas exploitation. This expansion is accompanied by a rise in wellbore instability and drilling accidents caused by downhole complexities [6,7]. During drilling operations, drilling fluid performance directly affects both operational efficiency and reservoir protection. This underscores the critical importance of employing high-performance drilling fluids in shale gas production [8].

Oil-based drilling fluids offer significant advantages, including superior inhibition, excellent lubrication, strong contamination resistance, and good thermal stability. These properties effectively inhibit shale hydration, reduce the wellbore collapse rate, and enhance wellbore stability [9,10]. However, such fluids are typically costly, pose environmental concerns, and their application requires careful management to mitigate potential formation damage [11]. In contrast, water-based drilling fluids are more environmentally friendly, easier to handle, and relatively inexpensive [12]. Yet, their performance is often prone to degradation under high-temperature and high-pressure (HTHP) conditions. This necessitates the development of improved water-based systems capable of withstanding severe downhole environments. To address fluid loss issues during drilling, researchers have incorporated various materials as fluid loss control agents and plugging agents into water-based drilling fluids [13,14,15].

Traditional inorganic plugging materials, such as nano-SiO_2_, graphene, and carbon nanotubes, offer significant practical advantages in leakage prevention and control due to their high strength and durability [16,17,18]. However, they generally suffer from poor dispersibility, a tendency to flocculate, limited deformability, and inadequate self-adaptability within formation environments. When the particle size does not closely match that of the leakage channels, these materials cannot effectively enter or penetrate deeper into the fractures through deformation, resulting in inefficient sealing [19]. To address these limitations, some researchers have combined the flexibility of polymers with the rigidity of inorganic particles via chemical modification [20]. For instance, Liu et al. developed a novel modified nano-silica/polymer composite, where the inorganic nano-SiO_2_ provides rigid structural support and the polymer chains contribute flexibility and improved dispersion. This composite offers an efficient and environmentally friendly nano-plugging solution for shale gas drilling [21]. Nevertheless, as oil exploration advances to more challenging environments, the requirements for plugging agents are becoming increasingly diverse and demanding.

The advancement of intelligent responsive materials has driven innovation across numerous fields. Several of these materials have garnered significant attention within the petroleum industry and found broad application. It is generally recognized that this type of thermosensitive hydrogel possesses abundant hydrophilic and hydrophobic groups. Temperature variation can alter the relative strength of hydrophobic association and hydrogen bonding within the system, disrupt the hydration structure between polymer chains and water molecules, induce substantial conformational changes in molecular chains, and further trigger abrupt macroscopic phase transition. From the perspective of thermodynamics and molecular mechanism, the core driving force behind the LCST phase transition originates from the entropy increase resulting from the release of confined water surrounding hydrophobic segments. This process is accompanied by the dissociation of polymer–water hydrogen bonds and the enhancement of interchain hydrophobic interactions [22,23,24]. Based on this principle, typical lower critical solution temperature (LCST) polymers include poly(N-isopropyl acrylamide) (PNIPAM) and poly(N-vinyl caprolactam) (PNVCL). To tailor the LCST or enhance specific properties, researchers commonly adjust the copolymer’s hydrophilic-to-hydrophobic balance by incorporating different hydrophilic and hydrophobic monomers.

Wei et al. [25] synthesized an intelligent temperature-sensitive composite by copolymerizing N-isopropylacrylamide (NIPAM) with both hydrophilic and hydrophobic monomers, such as N-hydroxyethyl acrylamide (HEAA). This material exhibits highly sensitive and reversible thermos-sensitivity and can form an effective barrier on shale surfaces at elevated temperatures, leading to a favorable plugging effect.

Zhu et al. [26] introduced NIPAM into the free-radical polymerization process with nano-silica, obtaining a temperature-sensitive polymer with suitable rheological properties for high-temperature and high-salinity environments. Wang et al. [27] graft-polymerized NIPAM and acrylic acid (AA) onto methyl methacrylate-styrene (MMA-ST) nanospheres to investigate the influence of the hydrophilic monomer ratio on the LCST. The resulting product also demonstrated effective plugging performance. Some researchers have imparted temperature sensitivity to bentonite by modifying it with NIPAM. After chemical modification, the bentonite displayed a temperature response alongside improved rheology and thermosensitive characteristics [28,29]. Xie et al. [30] copolymerized AM, 2-acrylamido-2-methylpropanesulfonic acid (AMPS), and N-vinylcaprolactam (NVCL) to create a temperature-sensitive rheological modifier. This modifier leverages the synergistic effect between high-temperature hydrophobic self-assembly and low-temperature bentonite interaction, effectively enhancing the rheological stability of drilling fluids across a broad temperature range (4–95 °C) and exhibiting thermal-thickening behavior.

The aforementioned thermosensitive polymers exhibit promising application potential in oil and gas development. Nevertheless, pure PNIPAM- and PNVCL-based synthetic thermosensitive polymers still present prominent limitations in practical applications, including poor biodegradability, inadequate environmental compatibility, weak performance in mud cake modification, and facile loss of thermos-responsive reversibility under complex formation conditions. Numerous studies have demonstrated that cellulose derivatives generally possess favorable hydrophilicity and moisture retention capacity. Incorporating cellulose derivatives into water-based drilling fluids can improve mud cake quality, as well as enhance the rheological behavior and thermal stability of the drilling fluid system [31,32]. With increasingly stringent regulations regarding environmentally friendly drilling fluids and growing emphasis on environmental sustainability globally, the development of readily biodegradable products using eco-friendly raw materials has attracted widespread attention worldwide. Research interest has also been increasingly directed toward stimulus-responsive materials derived from natural biological polysaccharides, represented by cellulose. Relevant investigations have demonstrated that chemical modification of cellulose derivatives can significantly expand their application scope [33,34]. Current research on modifying these materials for temperature sensitivity has garnered attention, suggesting considerable potential for their application in drilling and plugging operations [35,36,37].

Therefore, the HAAN temperature-sensitive plugging agent was synthesized via free-radical graft polymerization, wherein AM, ACMO, and NVP were grafted onto HPMC. This approach aimed to enhance its temperature and salt tolerance by introducing functional monomers to withstand complex downhole conditions. By optimizing the synthesis conditions and employing a series of characterization techniques to identify the most cost-effective formulation with superior performance, an optimal temperature-sensitive drilling fluid plugging agent was obtained. The developed plugging agent exhibited excellent sealing performance, which was comprehensively evaluated using conventional drilling fluid testing equipment.

## 2. Materials and Methods

### 2.1. Materials

Acrylamide (AM, AR), potassium persulfate (KPS, AR), sodium bisulfite (SHS, AR) purchased from Chengdu Kelong Chemical Co., Ltd. (Chengdu, China), 4-Acrylmorpholine (ACMO, 98%) was purchased from Maclin Biochemical Technology Co., Ltd. (Shanghai, China), hydroxypropyl methyl cellulose (type I HPMC, M_w_ ≈ 1.6 × 10^4^ Da, AR), the degrees of the methoxy group and hydroxypropyl group were 28–30% and 7.5–12%, N-vinyl-2-pyrrolidone (NVP) was provided by Aladdin Biochemical Technology Co., Ltd. (Shanghai, China), Na-bentonite was provided by Henan Yixiang New Material Co., Ltd. (Hebi, China), and all water used was deionized water.

### 2.2. Synthesis of HAAN

**Figure 1:**
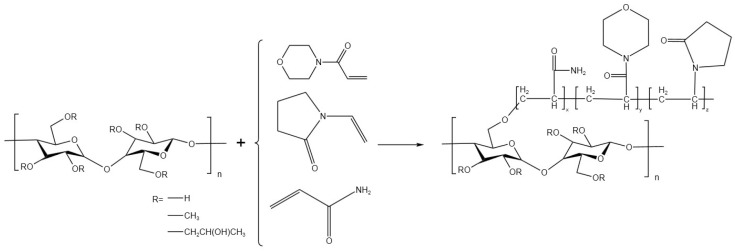
The synthetic route of HAAN.

Using HPMC, AM, ACMO, and NVP as reaction monomers, the polymer HAAN was prepared through free radical graft polymerization in an aqueous solution. The specific procedure is as follows: a certain proportion of HPMC was weighed and dissolved in deionized water. After stirring until completely dissolved, the solution was poured into a three-necked flask equipped with mechanical stirring and placed in a water bath. Nitrogen (N_2_) was purged through the solution for 30 min. The reaction temperature was set to 50 °C and stirring and nitrogen purging were continued. Once the temperature reached the reaction temperature, the pH of the solution was adjusted to 8–9. The reaction system employed a potassium persulfate/sodium bisulfite redox initiation system. After continuous initiation for 20 min, a pre-prepared aqueous solution of the monomers was gradually added dropwise into the solution over approximately 20 min. The reaction was allowed to proceed for a certain period. After the reaction was completed, the product was poured into an excess of acetone to precipitate. The precipitated product was cut into small pieces and soaked in acetone for purification, with the acetone solvent replaced regularly. Finally, the product was placed in a freeze dryer and dried for 2–3 days, after which it was ground to obtain the temperature-sensitive polymer powder HPMC-g-P(AM-ACMO-NVP). The reaction formula is shown in Figure 1 below. The grafting rate G was calculated using the following formula:

$$G=\frac{\left(W_{0}{-W}_{1}\right)}{W_{0}}\times 100\%$$

*W*_0_ and *W*_1_ represent the weights of HPMC and the graft copolymer, respectively.

### 2.3. Characterization

#### 2.3.1. Fourier Transform Infrared (FT-IR)

The structure of HAAN was analyzed by Fourier Transform Infrared Spectrometer. HAAN and potassium bromide were ground into powder at a mass ratio of 1:100, and then flakes were obtained using a lamination mechanism at a pressure of about 15–20 MPa. The flakes were placed in the sample bin for analysis by Fourier Transform Infrared Spectrometer (Nicolet 6700, Thermo Scientific Co., Waltham, MA, USA). The scanning range is 400 to 4000 cm^−1^, the scanning interval is 1.928 cm^−1^, and the scanning times are 32.

#### 2.3.2. ^1^H NMR Spectroscopic Characterization

An appropriate amount of HAAN was dissolved in 0.65 mL of heavy water D_2_O and placed in a nuclear magnetic tube. The temperature sensitive plugging agent was detected by Bruker AVANCE III HD400, Switzerland spectrometer.

#### 2.3.3. Thermogravimetric Analysis

The thermal stability of HAAN was tested by thermal analyzer (TGA/SDTA85/e, METTLER TOLEDO). During the test, a nitrogen atmosphere was maintained in the furnace through continuous purging; the heating rate was 10 °C/min, and the test temperature was 25~500 °C.

#### 2.3.4. Temperature-Sensitive Behavior Test

The temperature sensitivity of the polymer is determined by testing its light transmittance at different temperatures. When the temperature is higher than the phase transition temperature of the temperature-sensitive polymer, the temperature sensitive polymer will change from hydrophilic to hydrophobic. The LCST value of HAAN is determined by measuring the transmittance of HAAN at different temperatures by using an in situ variable temperature ultraviolet–visible spectrophotometer (PE Lambda 950) connected with a constant temperature trough. The wavelength range of UV–vis is 200–800 nm.

#### 2.3.5. High Temperature Rheological Property Test

The shear rate was set to 10 s^−1^ and the heating rate was 1 °C/min. The rheological measurements of the HAAN samples were performed using an Anton Paar MCR302 rheometer fitted with a 25 mm parallel-plate geometry, aiming to investigate the temperature dependence of HAAN viscosity. Then, the strain amplitude is fixed, and the test frequency is set to 1 Hz.

### 2.4. Conventional Performance Evaluation of Drilling Fluid

Deionized water, bentonite, and anhydrous sodium carbonate were weighed in a beaker according to the mass ratio of 100:4:0.16, stirred at a certain speed for 30 min, and hydrated at room temperature for 24 h to obtain bentonite-based slurry. The product was added to the base pulp and the aging test was carried out under the conditions of hot rolling at 130 °C and 160 °C for 16 h, respectively.

#### 2.4.1. API Filtration Performance Evaluation

The conventional properties of drilling fluids are evaluated according to the American Petroleum Institute (API) standard test program. The filtration loss (FL_API_) of drilling fluid treated with zwitterion filtration reduction agent HAAN before and after aging was measured by API Filtrate instrument (7NS-2A, Qingdao Tongchun Petroleum Instrument Co., Ltd., Qingdao, China). Test conditions: pressure 0.69 MPa at room temperature and filtration time 30 min. The high temperature and high-pressure filtration loss (FL_HTHP_) of drilling fluid before and after HAAN treatment was measured by high temperature and high-pressure (HTHP) Filtrate instrument (GGS42-2A, Haitongda, Qingdao, China). Test conditions: 160 °C, 3.45 Mpa pressure difference.

#### 2.4.2. Rheological Property Evaluation

The rheological properties of drilling fluid with HAAN were measured before and after aging by using the six-speed rotational viscometer (MK-03, Shandong Meke, Jinan, China). The apparent viscosity (AV), plastic viscosity (PV) and dynamic shear force (YP) of the drilling fluid were calculated according to API regulations. The formula is as follows:

(1) $$AV=\frac{1}{2}\Phi 600\left(mPa\cdot s\right)$$

(2) PV=Φ600−Φ300(mPa·s)

(3) YP=0.48 × (Φ300−PV)(Pa)

*Φ*300 and *Φ*600 are the readings of the six-speed rotational viscometer at 300 rpm and 600 rpm, respectively [38].

### 2.5. Mud Cake Morphology Analysis

The mud cake obtained from the base slurry and the base slurry containing 1 wt% HAAN was freeze-dried at room temperature and medium pressure, and the size of the mud cake was cut to 1 cm × 1 cm. All samples were sputter-coated with a thin gold layer to enhance electrical conductivity and imaging contrast. The surface morphology of the mud cake was examined using SEM (EV0 MA15, ZEISS International Co., Baden-Württemberg, Germany) operated at an accelerating voltage of 20 kV.

## 3. Results and Discussion

### 3.1. Optimization of Synthetic Conditions of HAAN

Various experimental conditions exert distinct influences on the product properties during the polymerization process. Considering that the concentration of HPMC (A), monomer ratio (B), total mass fraction of grafting monomers (C), reaction time (D), and initiator concentration (E) could affect the product performance and thereby the plugging efficiency in drilling fluid, an L_16_ (4^5^) orthogonal experimental design was employed to optimize the synthesis conditions. The fixed addition amount of 2 wt% and the high-temperature high-pressure fluid loss (FL_HTHP_, 130 °C, 3.45 MPa differential pressure) were used as evaluation criteria. The designed orthogonal array is presented in Table 1.

The orthogonal experimental results indicated that a larger R^X^ value reflects a greater influence of the corresponding factor on the experimental outcomes, whereas a larger *K*_*i*_^*X*^ value corresponds to poorer performance. As presented in Table 2, the order of factors affecting the FL_HTHP_ (130 °C) of HAAN, based on the R values, is A > B > C > D > E. In other words, the HPMC concentration exerts the most significant influence on the performance of the synthesized product, while the initiator concentration shows the least impact. It is hypothesized that an excessively high HPMC concentration may increase the solution viscosity, leading to uneven distribution of grafting sites and consequently a reduction in grafting efficiency. Taking into account both product performance and overall cost, the optimal synthesis conditions were determined as listed in Table 3. The grafting rate G of the as-synthesized product was 52%. Under these optimal conditions, the HAAN bentonite slurry exhibited a fluid loss of 7 mL at 130 °C, which is lower than that observed under any other tested condition.

### 3.2. Characterization of HAAN

#### 3.2.1. FTIR Analysis of HAAN

**Figure 2:**
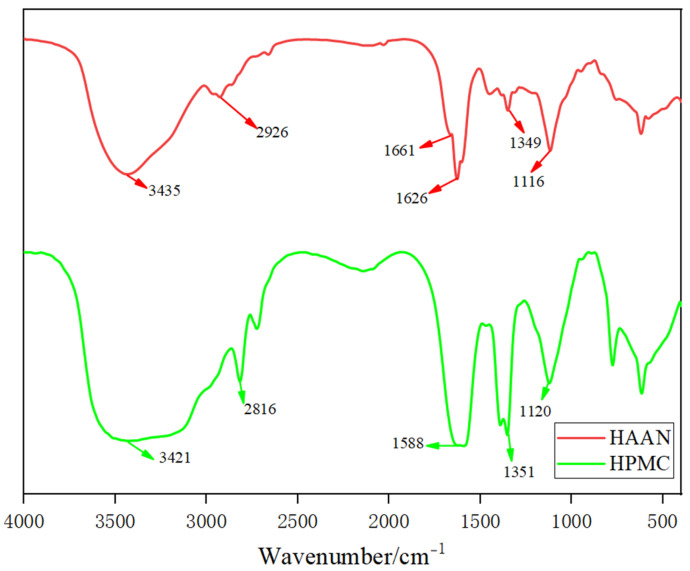
FT-IR spectrum of HAAN.

The FTIR spectra of HPMC and the synthesized HAAN are presented in Figure 2. In the HPMC spectrum, the broad absorption peak at 3421 cm^−1^ is attributed to the O-H stretching vibration. Peaks observed at 2816 cm^−1^ and 1351 cm^−1^ correspond to the C-H stretching vibration and the O-H bending vibration, respectively. The signal at 1588 cm^−1^ likely originates from residual bound water, while the distinct peak at 1120 cm^−1^ is assigned to the C-O stretching vibration. In contrast, the HAAN spectrum displays a broad overlapping band at 3435 cm^−1^, characteristic of combined O-H and N-H stretching vibrations. The peak at 2926 cm^−1^ is ascribed to the antisymmetric C-H stretching vibration. Notably, two new absorption peaks appear at 1661 cm^−1^ and 1626 cm^−1^, which are indicative of the carbonyl (C=O) stretching vibration in amide groups. Additional features include the C-H bending vibration of methylene (-CH_2_-) at 1349 cm^−1^ and the C-O skeletal stretching vibration at 1116 cm^−1^. The emergence of these characteristic absorptions, particularly the amide-related peaks, confirms the successful occurrence of graft polymerization.

#### 3.2.2. ^1^H NMR Analysis of HAAN

**Figure 3:**
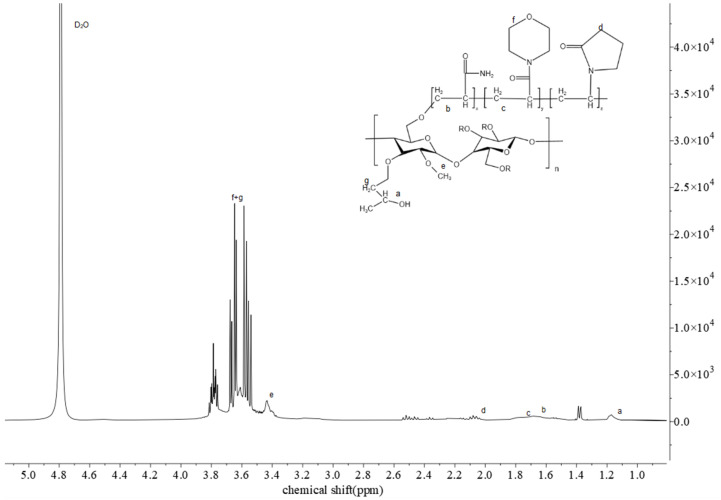
^1^H NMR spectrum of HAAN.

As shown in Figure 3, the ^1^H NMR spectrum of polymer HAAN reveals its characteristic proton resonances. The signal at 1.17 ppm corresponds to the hydrogen atoms of the methyl group (-(CH_3_)CH-). The methylene protons (-CH_2_-) on the polymer side chains give rise to signals at 1.67 ppm and 1.76 ppm. Resonances at 2.09 ppm and 2.20 ppm are assigned to the methyl group (-CH_3_CO-) and the methine group (-CHCO-) adjacent to carbonyl functionalities, respectively. A characteristic signal for the methoxy group (-CH_3_-O-) in the polymer backbone appears at 3.39 ppm. The complex multiplet in the region of 3.58–3.78 ppm is attributed to the protons of the morpholine ring (-N-CH_2_-CH_2_-O-) on the side chain and the methine protons in the hydroxypropyl groups of the main chain. Notably, proton signals from amino groups (-NH_2_-,-N-H-) are not observed due to deuterium exchange with the D_2_O solvent used for the measurement.

#### 3.2.3. Thermal Analysis of HAAN

**Figure 4:**
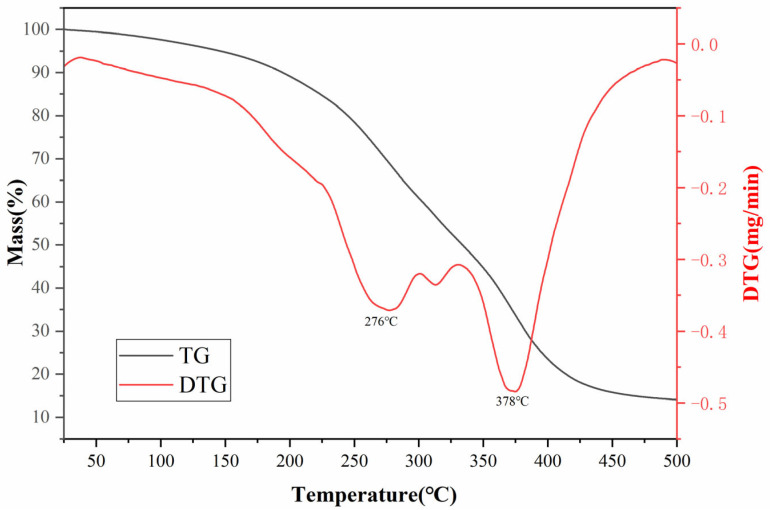
TG curves of the HAAN.

The thermogravimetric (TG) curve of HAAN is presented in Figure 4, revealing that its thermal decomposition occurs primarily in three distinct stages. The first stage (40–276 °C) involves the evaporation of free and bound water, accompanied by the decomposition of thermally labile amide groups within the polymer, resulting in a mass loss of approximately 30%. In the second stage (276–378 °C), a further mass loss of about 38% is observed. This stage is attributed to the ring-opening decomposition of the residual amide groups and the more stable morpholine ring structure, as well as partial cleavage of the polymer backbone, including the breakage of C-O and C-N bonds. During the final stage (>378 °C), the mass loss rate gradually decreases as the polymer undergoes carbonization, culminating in a final residual mass of approximately 15%. This remaining yield indicates that the polymerization product possesses moderate thermal stability.

#### 3.2.4. Temperature-Sensitive Behavior

**Figure 5:**
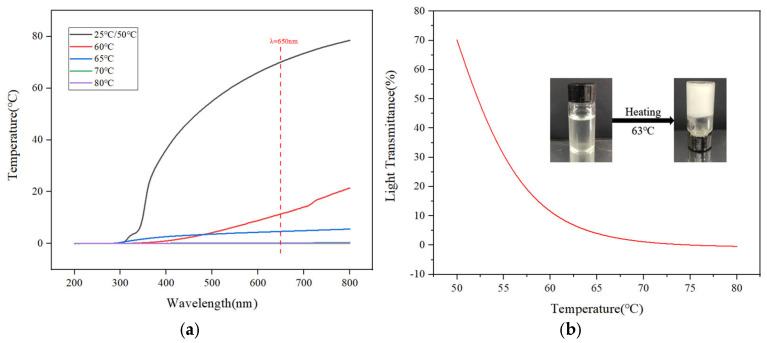
The transmittance of HAAN at different temperatures (**a**); transmittance curve at 650 nm at different temperatures (**b**).

The thermosensitive behavior of HAAN was investigated by measuring its light transmittance (λ = 200–800 nm) at varying temperatures, as shown in Figure 5. The results demonstrate that the transmittance of HAAN decreases with increasing temperature. A notable decrease occurs when the temperature reaches 60 °C, and the trend continues during subsequent heating until stabilizing after 70 °C, indicating a distinct thermal response. At this point, the sample has completely transformed into a non-flowing gel. This behavior is attributed to the amphiphilic nature of the HAAN molecular chains, which contain both hydrophilic and hydrophobic moieties. Below the lower critical solution temperature (LCST), hydrophilic groups such as amides and hydroxyls form strong hydrogen bonds with water molecules, leading to full hydration and chain expansion of the polymer, thereby resulting in a transparent sol state. Above the LCST, hydrogen bonding is weakened, exposing hydrophobic groups (e.g., methoxy groups) along the macromolecular chains. Consequently, hydrophobic associations are enhanced, promoting the formation of a three-dimensional network structure and macroscopic gelation. As observed in Figure 5b, when heated from 1 °C/min to 63 °C, the sol transforms into a solid-like gel that remains stable upon inversion of the test tube.

According to the method previously reported [39,40], by plotting the light transmittance (%) of the polymer at 650 nm as a function of temperature, a four-parameter Logistic curve (Figure 5b) was used to fit the curve (R^2^ > 0.99). The formula used was as follows:

$$y=\left[\frac{0.79789-0.20701}{1+{\left(\frac{x}{81.55445}\right)}^{2.7885}}+0.20701\right]\times 100\%$$

where *y* is the transmittance (%) of HAAN; *x* is the temperature (°C).

The phase transition temperature can be confirmed by the inflection point of the S curve of transmittance versus temperature at a specific wavelength of 650 nm [41], and the temperature response point of HAAN is about 63 °C as shown in Figure 5b.

#### 3.2.5. High-Temperature Rheological Analysis

**Figure 6:**
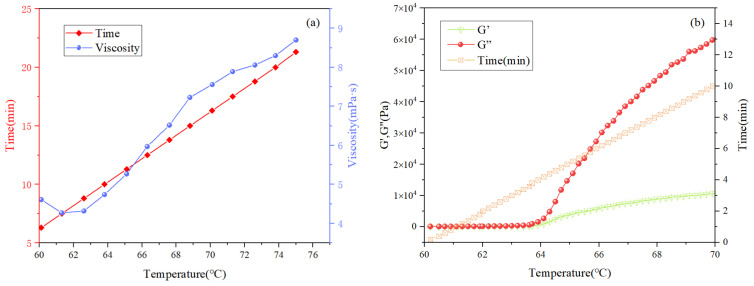
Viscosity-temperature test (**a**); and rheological property test of HAAN (**b**).

Rheological measurements before and after the phase transition confirmed that the HAAN gel undergoes a temperature-induced sol–gel transition, accompanied by a substantial increase in gel strength. As shown in Figure 6a, the viscosity of the HAAN gel initially decreased and then gradually increased with rising temperature. From the viscoelastic test results, it can be observed that when the temperature reached 60 °C, the storage modulus (G′) began to exceed the loss modulus (G″), indicating a gradual shift toward elastic-dominated behavior. A sharp increase in both moduli occurred at approximately 63 °C, after which they leveled off, suggesting that the gelation process was complete and had reached a steady state.

#### 3.2.6. Temperature Tolerance Test

**Figure 7:**
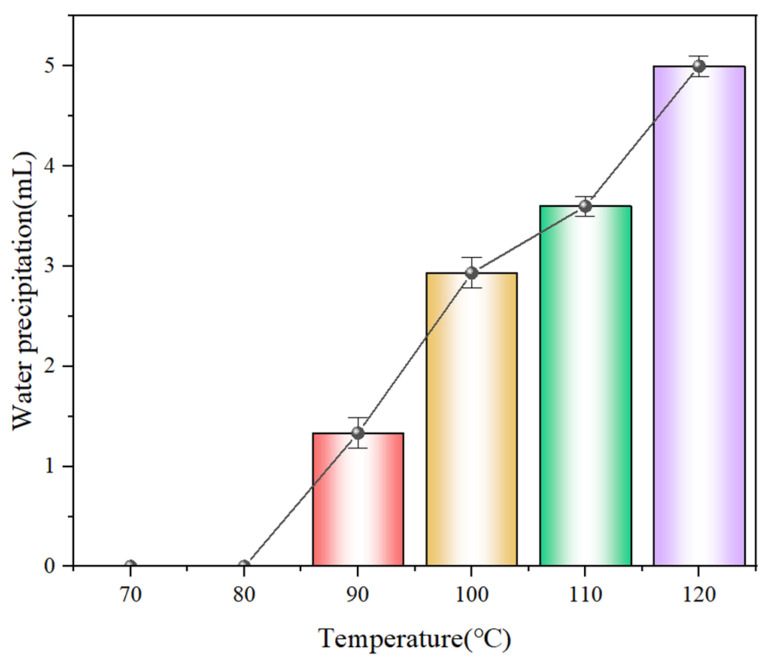
HAAN transmittance curve at different temperatures.

To evaluate the thermal stability of HAAN under simulated formation conditions, the gel was aged for 24 h at various temperatures, and its stability was assessed based on the extent of water syneresis after aging. As shown in Figure 7, no water separation was observed after aging at 70 °C and 80 °C for 24 h, indicating stable gel performance. At 90 °C, a trace amount of water separation occurred. When the temperature exceeded 100 °C, noticeable solid–liquid separation took place; however, after water loss, the gel contracted and exhibited increased mechanical strength, demonstrating good heat resistance. Even after heating to 160 °C, the material retained its gel morphology, confirming its potential applicability in high-temperature downhole environments.

### 3.3. Seal Performance of HAAN Plugging Agent

#### 3.3.1. Rheological Property Analysis and Filtration Property Analysis

**Figure 8:**
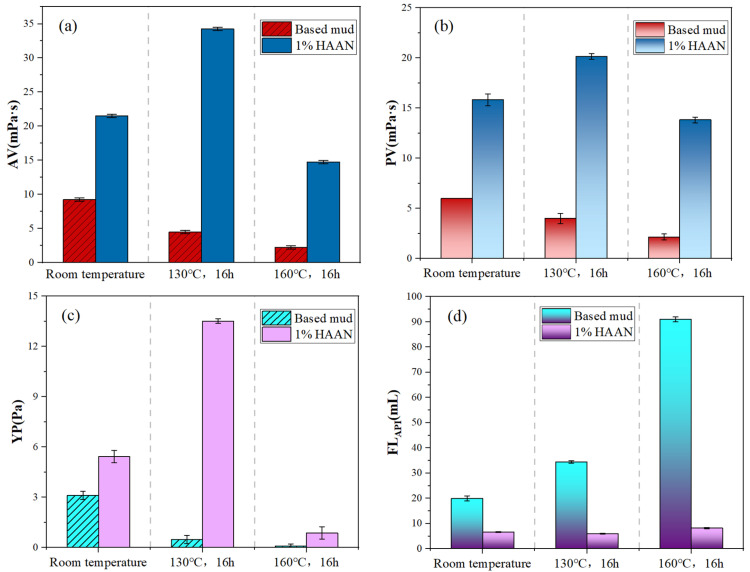
Apparent viscosity (**a**), plastic viscosity (**b**), dynamic shear force, (**c**) and FL_API_ (**d**) values of Na-Bent containing HAAN before and after aging for 16 h at 160 °C.

As shown in Figure 8, the addition of HAAN to the base slurry resulted in increases in the AV, PV and YP compared to the untreated blank slurry. As a long-chain polymeric compound, HAAN can extend its molecular chains when dissolved in aqueous drilling fluid. Through a combination of chain entanglement, hydration, and steric-hindrance effects, this additive provides effective viscosity enhancement and improves cuttings-lifting capacity, thereby enhancing the overall suspension and cuttings-carrying performance of the drilling fluid. After high-temperature aging, the rheological properties of the base slurry decreased substantially. In contrast, although some fluctuation was observed, the key rheological parameters of the slurry containing HAAN remained significantly higher than those of the aged base slurry. Furthermore, the API filtration loss of the bentonite base slurry was reduced from 21 mL to 6.6 mL. After aging, the filtration loss decreased from 92 mL to 8 mL, representing a reduction of approximately 91% compared to the aged base slurry. The fluid loss performance is superior to those of water-based drilling fluids containing polyanionic cellulose (PAC, 66.7%) and Fe_3_O_4_-modified CMC (65.2%) [42,43].

**Figure 9:**
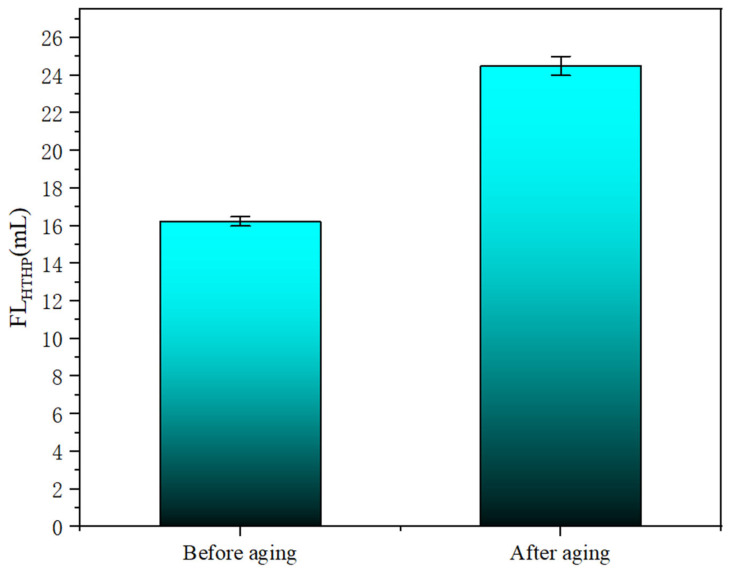
FL_HTHP_ of drilling mud containing HAAN before and after aging at 160 °C.

Figure 9 shows the FL_HTHP_ results of adding HAAN drilling fluid. The base mud alone failed to form an effective filter cake under high-temperature, high-pressure conditions, and its 30 min filtration loss could not be measured. In contrast, the drilling fluid containing 1 wt% HAAN exhibited an FL_HTHP_ of only 18 mL. Even after aging at 160 °C, the FL_HTHP_ remained as low as 24 mL. This improvement in filtration control can be attributed to the thermosensitive behavior of HAAN. When the temperature rises, hydrophobic groups along the polymer backbone become more exposed, enhancing hydrophobic association. Concurrently, these associations cause the molecular chains to contract and form aggregates, which promote the formation of a dense, low-permeability filter cake, thereby effectively reducing fluid filtration.

#### 3.3.2. Salt Tolerance Test

In bentonite slurry, high concentrations of salt ions strongly compress the diffuse double layer on the surfaces of clay particles and weaken the electrostatic repulsion between them. Consequently, the particles tend to flocculate primarily via “face-edge” or “edge-edge” association, forming coarse, loose aggregates. The resulting filter cake is characterized by coarse pores, a loose structure, and high permeability, which fails to effectively retain free water. This typically leads to deteriorated rheological properties and a rapid increase in filtration loss.

As shown in Table 4, HAAN exhibited excellent salt tolerance across a wide range of salinities (NaCl ≤ 100,000 mg/L, CaCl_2_ ≤ 5000 mg/L, and KCl ≤ 20,000 mg/L). The apparent viscosity, plastic viscosity, and yield point of the slurry containing HAAN—both before and after aging—remained superior to those of the base slurry at all tested salt concentrations. Furthermore, the filtration loss showed no marked change under these conditions, increasing only slightly. These results indicate that HAAN effectively mitigates the structural damage caused by salts to bentonite, enabling it to maintain effective fracture-sealing and filtration-control capabilities within the tested salinity range.

#### 3.3.3. Morphology Analysis of Mud Cake

**Figure 10:**
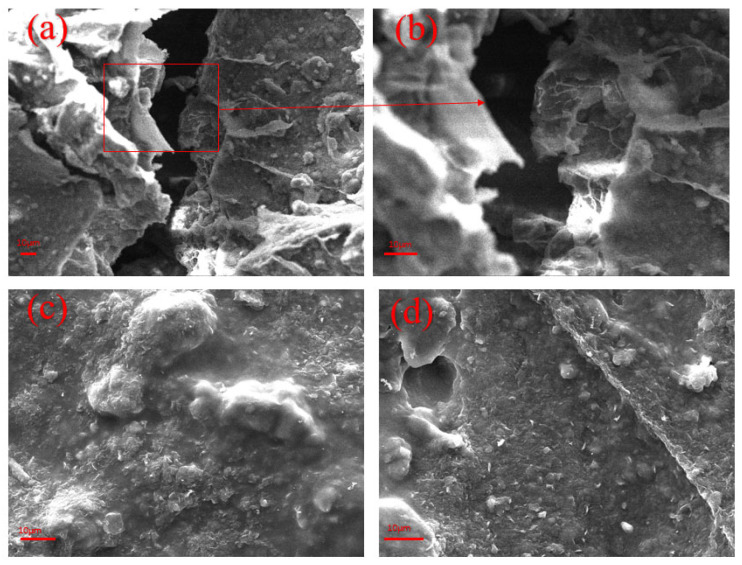
Micromorphology of filter cake before and after polymer treatment: (**a**,**b**) blank base slurry; (**c**) HAAN-treated mud cake at room temperature; (**d**) HAAN-treated mud cake at 160 °C.

Under the pressure differential between the wellbore fluid column and the formation, free water from the drilling fluid infiltrates the formation, while solid particles are retained on the wellbore wall, gradually accumulating to form a filter cake. A high-quality filter cake should exhibit rapid formation, compactness, and toughness to effectively seal formation pores and micro-fractures, significantly reduce total filtration loss, and maintain wellbore stability. Figure 10 presents the surface morphology of API filter cakes from bentonite mud and bentonite mud containing HAAN, both before and after aging. As shown in Figure 10a,b, the surface of the pure bentonite mud cake is rough and exhibits numerous cracks, through which free water can readily pass, resulting in high filtration loss under high differential pressure. In contrast, Figure 10c,d display the microstructure of the filter cake from HAAN-containing bentonite mud before and after aging at 160 °C. It is evident that the addition of HAAN yields a much denser filter cake with fewer pores and no obvious cracks, both before and after aging. A compact film forms on the surface, which substantially enhances the filter-cake strength. This improved structure effectively mitigates free-water invasion and helps maintain wellbore stability in high-temperature environments.

## 4. Conclusions

In this study, a temperature-sensitive graft copolymer HAAN was successfully synthesized via free-radical polymerization. As a fluid-loss additive in water-based drilling fluids, it demonstrates excellent performance. Structural characterization by FTIR and ^1^H NMR confirmed the successful grafting of monomers and the formation of the target product. The copolymer exhibits distinct thermosensitive phase-transition behavior, transforming from a free-flowing sol to a non-flowing gel state upon heating. Furthermore, the agent shows outstanding plugging efficiency under high-temperature conditions, along with notable thermal stability and salt tolerance. After aging at 160 °C, the filtration loss was reduced by approximately 91%. The additive also maintains favorable rheological properties and provides effective sealing within a wide salinity range (NaCl ≤ 10,000 mg/L, CaCl_2_ ≤ 5000 mg/L, or KCl ≤ 2000 mg/L), indicating good adaptability to complex downhole environments. Additionally, this study offers a new perspective for developing environmentally friendly drilling-fluid plugging agents, suggesting promising application prospects. Future research will focus on optimizing its performance under harsh drilling conditions, evaluating its field compatibility, and conducting pilot-scale trials to facilitate the practical application of cellulose-based smart drilling fluid additives.

## Figures and Tables

**Table 1:** Factors and levels of L_16_ (4^5^) orthogonal test.

Factor					
Level	A	B	C	D	E
1	A1 (1%)	B1 (5:3:2)	C1 (1:0.5)	D1 (2 h)	E1 (0.5%)
2	A2 (3%)	B2 (6:2:2)	C2 (1:1)	D2 (3 h)	E2 (1%)
3	A3 (5%)	B3 (4:3:3)	C3 (1:1.5)	D3 (4 h)	E3 (1.5%)
4	A4 (7%)	B4 (4:4:2)	C4 (1:2)	D4 (5 h)	E4 (2%)

**Table 2:** Analysis of the results of L_16_ (4^5^) orthogonal test.

Test No.	A	B	C	D	E	FL_HTHP_ (130 °C)
1	1	1	1	1	1	33
2	1	2	2	2	2	43
3	1	3	3	3	3	30
4	1	4	4	4	4	69
5	2	1	2	3	4	7
6	2	2	1	4	3	20.5
7	2	3	4	1	2	17
8	2	4	3	2	1	21.5
9	3	1	3	4	2	18.5
10	3	2	4	3	1	59
11	3	3	1	2	4	27
12	3	4	2	1	3	25
13	4	1	4	2	3	11
14	4	2	3	1	4	28
15	4	3	2	4	1	15.5
16	4	4	1	3	2	18.5
K_1_	43.75	17.375	24.75	32.25	25.75	
K_2_	16.5	37.625	22.625	24.25	25.625	
K_3_	32.375	22.375	24.5	21.625	28.625	
K_4_	18.25	33.5	39	32.75	30.875	
R	27.25	20.25	16.375	11.125	5.25	

$K_{i}^{X}=(\sum the\;{FL}_{HTHP}\left(160\;{}^{\circ}C\right)\;value\;at\;X_{i})/4.$ $R_{i}^{X}=max\left\{K_{i}^{X}\right\}-min\left\{K_{i}^{X}\right\}.$ Note: X can be A, B, C, D, E, and i can be 1, 2, 3, 4.

**Table 3:** Optimal synthesis conditions.

Concentration of HPMC	Monomer Ratio	Total Mass Fraction of Grafting Monomers	Reaction Time	Initiator Concentration	FL_HTHP_ (130 °C)
3%	5:3:2	1:1	4 h	1%	7

**Table 4:** Median particle size and volume average particle size of drilling fluid system.

Drilling Fluid System	Condition	AV (mPa·s)	PV (mPa·s)	YP (Pa)	FL_API_ (mL)
Based mud	Before aging	9	6	3	21
	160 °C, 16 h	2.5	2	0.24	92
Based mud + 1%HAAN	Before aging	21	15.5	3	6.6
	160 °C, 16 h	16	14	1	8
1%HAAN + 2.5%NaCl	Before aging	26	19	7	6
	160 °C, 16 h	18	14	4	6.3
1%HAAN + 5%NaCl	Before aging	29	19	10	5
	160 °C, 16 h	21.5	17	9	7
1%HAAN + 7.5%NaCl	Before aging	26	13	13	6.1
	160 °C, 16 h	25	24	1	9.8
1%HAAN + 10%NaCl	Before aging	43.5	29	14	6
	160 °C, 16 h	13	13	0	15
1%HAAN + 0.5%CaCl_2_	Before aging	25.5	18	7	6.3
	160 °C, 16 h	30	22	7.5	6.8
1%HAAN + 1%KCl	Before aging	35.5	25	10	7.2
	160 °C, 16 h	29.5	23	6	10
1%HAAN + 2%KCl	Before aging	35	21	13	7.6
	160 °C, 16 h	22.5	18	4	13.1

## Data Availability

The data presented in this study are available on request from the corresponding author.
